# Correlations among adiposity measures in school-aged children

**DOI:** 10.1186/1471-2431-13-99

**Published:** 2013-06-24

**Authors:** Caroline E Boeke, Emily Oken, Ken P Kleinman, Sheryl L Rifas-Shiman, Elsie M Taveras, Matthew W Gillman

**Affiliations:** 1 Department of Epidemiology, Harvard School of Public Health, 677 Huntington Avenue, Boston, MA 02115, USA; 2 Department of Nutrition, Harvard School of Public Health, 677 Huntington Avenue, Boston, MA 02115, USA; 3 Channing Division of Network Medicine, Brigham and Women’s Hospital, 181 Longwood Avenue, Boston, MA 02115, USA; 4 Department of Population Medicine, Obesity Prevention Program, Harvard Medical School and Harvard Pilgrim Health Care Institute, 133 Brookline Avenue, 3rd Floor, Boston, MA 02215, USA

**Keywords:** Adiposity, Obesity, DXA, BMI

## Abstract

**Background:**

Given that it is not feasible to use dual x-ray absorptiometry (DXA) or other reference methods to measure adiposity in all pediatric clinical and research settings, it is important to identify reasonable alternatives. Therefore, we sought to determine the extent to which other adiposity measures were correlated with DXA fat mass in school-aged children.

**Methods:**

In 1110 children aged 6.5-10.9 years in the pre-birth cohort Project Viva, we calculated Spearman correlation coefficients between DXA (n=875) and other adiposity measures including body mass index (BMI), skinfold thickness, circumferences, and bioimpedance. We also computed correlations between lean body mass measures.

**Results:**

50.0% of the children were female and 36.5% were non-white. Mean (SD) BMI was 17.2 (3.1) and total fat mass by DXA was 7.5 (3.9) kg. DXA total fat mass was highly correlated with BMI (r_s_=0.83), bioimpedance total fat (r_s_=0.87), and sum of skinfolds (r_s_=0.90), and DXA trunk fat was highly correlated with waist circumference (r_s_=0.79). Correlations of BMI with other adiposity indices were high, e.g., with waist circumference (r_s_=0.86) and sum of subscapular plus triceps skinfolds (r_s_=0.79). DXA fat-free mass and bioimpedance fat-free mass were highly correlated (r_s_=0.94).

**Conclusions:**

In school-aged children, BMI, sum of skinfolds, and other adiposity measures were strongly correlated with DXA fat mass. Although these measurement methods have limitations, BMI and skinfolds are adequate surrogate measures of relative adiposity in children when DXA is not practical.

## Background

Gold standard measures of adiposity such as the four-compartment model, which calculates adiposity using independent measurements of total body water, body density, and bone mass [1] are not feasible in large epidemiologic studies. Even dual x-ray absorptiometry (DXA), which performed very well against the gold standard in children ages 6 to 18 years [2], is not always available or feasible, and it emits radiation, which though a small amount, may discourage subjects. Therefore, it is important to identify reasonable alternative methods that are highly correlated with gold standard measures, and to understand how these methods correlate with each other.

Body mass index (BMI) and waist circumference measure overall and central adiposity, respectively, relatively well in adults [3]. Fewer studies have compared correlations of multiple anthropometric measures with DXA in children [4-7]. In 75 3-8-year-old white and Hispanic American children, the correlation of BMI with DXA total fat mass was 0.85, and the correlation of waist circumference with DXA trunk fat was 0.84 [4]. Correlations between BMI or BMI z-score and DXA percent body fat were similar in two larger studies in youth [8,9]. In other studies in children and adolescents, higher BMI and waist circumference were associated with metabolic risk factors for chronic disease [10-13]; for example, in the Avon Longitudinal Study of Parents and Children among 7589 children ages 8.8-11.7 years, BMI was correlated with systolic blood pressure (r=0.42), triglycerides (r=0.22), C-reactive protein (r=0.41), and interleukin-6 (r=0.23) [13]. However, BMI does not explicitly distinguish between fat and lean mass, so there are still concerns about its use in pediatric studies. Another low cost and safe option for measuring adiposity is skinfold thickness. If not accompanied by rigorous training, however, intra- and inter-rater reliability can be low [14,15]. Bioelectrical impedance is another option that was well-correlated with DXA in previous studies in children (r>0.7), but it requires expensive equipment and may underestimate fat mass and overestimate fat-free mass [4,16-18].

While some previous studies have examined correlations of adiposity measurement methods in children, most were relatively small, spanned a large age range, and/or were limited to only a few measures of adiposity. We sought to examine the extent to which measures of total and central adiposity, including BMI, circumferences, skinfolds, bioimpedance, and DXA fat mass, were correlated with each other in a large study population of multiethnic school-aged children.

## Methods

### Subjects/study design

We studied 1110 children in Project Viva, an ongoing prospective pre-birth cohort study initiated in 1999. Women joined the study during their first prenatal visit at Harvard Vanguard Medical Associates, a large multi-specialty group practice in eastern Massachusetts. Eligibility criteria included fluency in English, gestational age less than 22 weeks at first prenatal visit, and singleton pregnancy. Additional details of recruitment and retention procedures have been published elsewhere [19]. After obtaining informed consent, we performed in-person study visits with both mothers and children. The mean (SD, range) child age was 7.9 (0.8, 6.5-10.9) years at the study visit. The institutional review board of Harvard Pilgrim Health Care Institute approved the study protocols.

### Measurements

At the in-person visit, trained research assistants measured height to the nearest 0.1 cm using a calibrated stadiometer (Shorr Productions, Olney, Maryland) and weight to the nearest 0.1 kg using a calibrated scale (Tanita model TBF-300A, Tanita Corporation of America, Inc., Arlington Heights, IL). We computed each child’s BMI using the following formula: BMI=weight/height^2^ (kg/m^2^). We calculated age-sex-adjusted BMI z-score and BMI percentile using the 2000 Centers for Disease Control and Prevention reference data [20].

We measured subscapular (SS) and triceps (TR) skinfold thicknesses to the nearest 0.1 mm using Holtain calipers (Holtain Ltd, Crosswell, Wales) and calculated the sum (SS + TR) and ratio (SS:TR) of the two thicknesses. The correlations of other measures of adiposity with subscapular or triceps thickness individually were very similar to the correlations with sum of the two, so we chose to show results for only sum of skinfolds. We measured hip and waist circumferences to the nearest 0.1 cm using a Hoechstmass measuring tape (Hoechstmass Balzer GmbH, Sulzbach, Germany), and calculated waist-to-hip circumference ratios. We measured middle upper arm circumference using a Ross measuring tape (Ross Products Division, Abbott Laboratories Inc., Columbus, OH).

Research assistants performing the measurements followed standardized techniques [21] and participated in biannual in-service training to ensure measurement validity. Inter- and intra-rater measurement errors were within published reference ranges for all of the measurements [22]. Experienced field supervisors provided ongoing quality control by observing and correcting measurement technique every 3 months.

We measured bipolar bioelectrical impedance using a Tanita scale model TBF-300A (Tanita Corporation of America, Inc., Arlington Heights, IL) foot-to-foot body composition analyzer. We calculated fat mass and fat-free mass indices for DXA and bioelectrical impedance measurements using the following formula: (mass in kg)/(height in meters)^2^.

Trained research assistants performed whole body DXA scans on the children (n=875) using a Hologic model Discovery A (Hologic, Bedford, MA) that they checked for quality control daily by scanning a standard synthetic spine to check for machine drift. We used Hologic software QDR version 12.6 for scan analysis. A single trained investigator (CEB) checked all scans for positioning, movement, and artifacts, and defined body regions for analysis. Intrarater reliability on duplicate measurements was high (r=0.99).

### Statistical analysis

We calculated Spearman correlation coefficients between pairs of adiposity measurements. We chose to focus on Spearman correlation coefficients because they do not assume normality and are conservative when linearity holds. However, we also ran Pearson correlations on natural log-transformed variables for comparison. We also examined correlations for boys and girls separately. We additionally calculated correlation coefficients among lean body mass measurements. Since children varied in exact age at study visit and measures differed by sex, we also calculated correlations adjusted for exact age at study visit and sex, but this adjustment did not substantially change correlations. Therefore we show only unadjusted correlations. We created Bland-Altman plots to assess the extent to which agreement between BMI and DXA fat mass index varied by amount of adiposity.

To assess whether measuring sum of skinfolds in addition to BMI improved associations with fat mass, we conducted linear regression of DXA fat mass index on BMI alone vs. BMI and sum of skinfolds. We compared the R-square values from the two models to assess the proportion of the variance in DXA fat mass explained by these measures.

We computed the difference between mean values of bioimpedance and DXA measures of fat mass and fat- free mass to assess any absolute difference between these measurement methods.

To assess how well BMI-defined obesity detects elevated DXA fat mass, we identified the children with BMI **≥**95^th^ age- and sex-specific percentile (“obesity”) and **≥**85^th^ percentile (“overweight plus obesity”) according to reference data from the Centers for Disease Control and Prevention [20]. We defined DXA percent body fat cutpoints for “obesity” and “overweight plus obesity” to generate the same percentage when determined by BMI percentile among subjects with both BMI and DXA available. The cutoffs were ≥30.10% body fat in females and ≥24.63% in males for “overweight plus obesity” and ≥34.00% body fat in females and ≥29.00% in males for “obesity”. We assessed the kappa statistic, sensitivity, and specificity, and the area under the receiver operating characteristic (ROC) curve. We calculated likelihood ratio positive as sensitivity/(1-specificity) and likelihood ratio negative as (1-sensitivity)/specificity.

We conducted a complete case analysis. We repeated the analysis using multiple imputation and 95% of the adiposity correlations examined were within 0.02, so for simplicity we decided to use the complete case approach.

We conducted all analyses using SAS version 9.2 or higher (Cary, NC).

## Results

Among the 1110 children in this analysis, 63.5% were white, 16.9% black, 4.2% Hispanic, 3.4% Asian, and 11.9% multiracial or other race/ethnicity, and 50.0% were female. Mean (SD) BMI was 17.2 (3.1) and BMI z-score was 0.4 (1.0). Bioelectrical impedance underestimated absolute fat mass (mean difference: -1.4 kg, 95% CI: -1.5, -1.4) and overestimated fat-free mass (mean difference: 1.1 kg, 95% CI: 1.0, 1.2) compared to DXA. Table 1 shows participant characteristics by normal weight, overweight, and obese categories.

**Table 1:** **Characteristics and adiposity measurements among 1110 Project Viva participants at ages 6.5-10.9 years**^**1**^

**Variable**	**N**	**Normal weight (n=826)**	**Overweight (n=149)**	**Obese (n=135)**
		**Mean (SD)/%**		
**Mother/Family**				
Smoked during pregnancy	1069	8.8%	7.1%	20.3%
Completed college	1104	71.3%	70.3%	45.9%
Multiparous	1110	51.7%	52.4%	56.3%
Annual household income > $70,000	1006	66.0%	69.3%	42.3%
Prepregnancy BMI (kg/m^2^)	1104	23.8 (4.5)	25.9 (5.2)	28.8 (7.3)
**Child**				
Female	1110	49.5%	54.4%	48.2%
Race/ethnicity	1108			
Asian		4.0%	3.4%	0.0%
Black		13.7%	15.4%	37.8%
Hispanic		3.5%	4.0%	8.9%
White		67.2%	66.4%	37.8%
More than one race/other		11.5%	10.7%	15.6%
Height (cm)	1110	127.6 (7.2)	130.9 (8.4)	134.0 (8.3)
Weight (kg)	1110	26.0 (4.1)	32.8 (5.6)	43.0 (10.5)
BMI (kg/m^2^)	1110	15.9 (1.2)	19.0 (1.0)	23.6 (3.5)
BMI z-score	1110	−0.05 (0.73)	1.31 (0.17)	2.07 (0.28)
Leg Length	1109	59.7 (4.6)	61.3 (5.5)	63.3 (5.8)
Waist circumference (cm)	1106	56.6 (4.1)	64.5 (5.0)	76.1 (9.4)
Hip circumference (cm)	1094	65.3 (4.7)	72.7 (6.0)	82.5 (8.5)
Subscapular skinfold thickness (mm)	1103	6.3 (2.0)	10.7 (7.5)	18.7 (7.2)
Triceps skinfold thickness (mm)	1106	9.6 (2.8)	14.5 (3.6)	20.6 (5.3)
BIA fat mass (kg)	1109	4.3 (1.7)	7.8 (2.3)	14.5 (5.9)
BIA % fat	1109	16.2 (4.3)	23.6 (4.2)	32.7 (6.5)
BIA fat-free mass (kg)	875	21.7 (3.1)	25.0 (3.9)	28.2 (5.3)
DXA total fat mass (kg)	875	5.8 (1.6)	9.1 (2.2)	15.0 (4.8)
DXA trunk fat (kg)	875	1.8 (0.6)	3.1 (1.0)	5.8 (2.3)
DXA % fat	875	22.2 (4.5)	27.8 (4.4)	34.8 (5.2)
DXA fat-free mass (kg)	875	20.3 (3.2)	23.7 (3.8)	27.5 (5.4)

^1^ Abbreviations as follows: *BIA* bioelectrical impedance. Normal weight, overweight, and obese defined as BMI z-score <85th percentile, 85th-<95th percentile, and ≥95th percentile, respectively.

Table 2 shows Spearman correlations among adiposity measures. DXA total fat mass was highly correlated with BMI [Spearman r (r_s_)=0.83; Pearson r (r_p_)=0.88], sum of skinfolds (r_s_=0.90; r_p_=0.93), and bioelectrical impedance body fat (r_s_=0.87; r_p_=0.89). DXA total fat mass was also highly correlated with middle upper arm circumference (r_s_=0.87). DXA fat mass index was also highly correlated with BMI (r_s_=0.80). Correlations of BMI with other adiposity indices were also high, for example with middle upper arm circumference (r_s_ =0.91) and sum of skinfolds (r_s_=0.79). All other correlations of these measures with each other were >0.78. DXA trunk fat was highly correlated with waist circumference (r_s_=0.79) but correlations with waist-to-hip and subscapular-to-triceps skinfold ratios were low (r_s_=0.20 and r_s_=0.16, respectively). Together, BMI and sum of skinfolds explained 89% of the variance of DXA fat mass index, as compared to 78% of the variance explained by BMI alone. Results were similar for BMI z-score. The Bland-Altman plot of BMI and fat mass index showed random scatter, indicating that the methods have good agreement at all levels of adiposity (plot not shown).

**Table 2:** **Spearman correlations among fatness indices at 6.5-10.9 years**^**1**^

	**BMI**	**BMI Z**	**Ht**	**Wt**	**Wt: ht**	**Waist circ**	**Waist: hip**	**SS: TR**	**SS+TR**	**BIA % fat**	**BIA fat**	**DXA trunk fat**	**DXA % fat**	**DXA fat**
	**Spearman R**													
**BMI**	1.00	0.98	0.38	0.84	0.93	0.86	0.23	0.22	0.79	0.81	0.88	0.81	0.63	0.83
**BMI Z**		1.00	0.28	0.77	0.88	0.81	0.26	0.21	0.76	0.81	0.85	0.78	0.63	0.80
**Height**			1.00	0.80	0.66	0.56	−0.07	0.15	0.33	0.33	0.53	0.44	0.14	0.47
**Weight**				1.00	0.98	0.87	0.11	0.23	0.69	0.70	0.86	0.76	0.49	0.80
**Wt:ht**					1.00	0.90	0.16	0.23	0.75	0.77	0.90	0.81	0.56	0.84
**Waist circ**						1.00	0.46	0.23	0.73	0.75	0.85	0.79	0.59	0.81
**Waist: hip**							1.00	0.19	0.19	0.25	0.20	0.20	0.21	0.17
**SS: TR**								1.00	0.14	0.18	0.21	0.16	0.08	0.13
**SS+TR**									1.00	0.80	0.81	0.89	0.84	0.90
**BIA % fat**										1.00	0.95	0.82	0.73	0.84
**BIA fat**											1.00	0.84	0.67	0.87
**DXA trunk fat**												1.00	0.89	0.98
**DXA % fat**													1.00	0.89
**DXA fat**														1.00

^1^Abbreviations are as follows: *Wt* weight, *ht* height, *BMI Z* age-sex-adjusted BMI z-score, *waist circ* waist circumference, *hip circ* hip circumference, *SS* subscapular skinfold thickness, *TR* triceps skinfold thickness, *BIA* bioelectrical impedance. Data from 1110 participants in Project Viva. Some participants did not have data for all forms of measurement, so some correlations were among as few as 873 participants.

Correlations were slightly higher in females than in males, although differences were unlikely to be clinically meaningful. For example, the correlation between BMI and DXA fat total fat was 0.84 for boys and 0.89 for girls, while the correlation between DXA and sum of skinfolds was 0.88 for boys and 0.90 for girls. The correlation between waist circumference and DXA trunk fat was 0.79 for boys and 0.87 for girls.

Correlations were stronger in black and Hispanic children than in whites (e.g. for BMI vs. DXA fat mass, r_s_=0.91 for blacks, 0.94 for Hispanics, 0.79 for whites).

**Figure 1:**
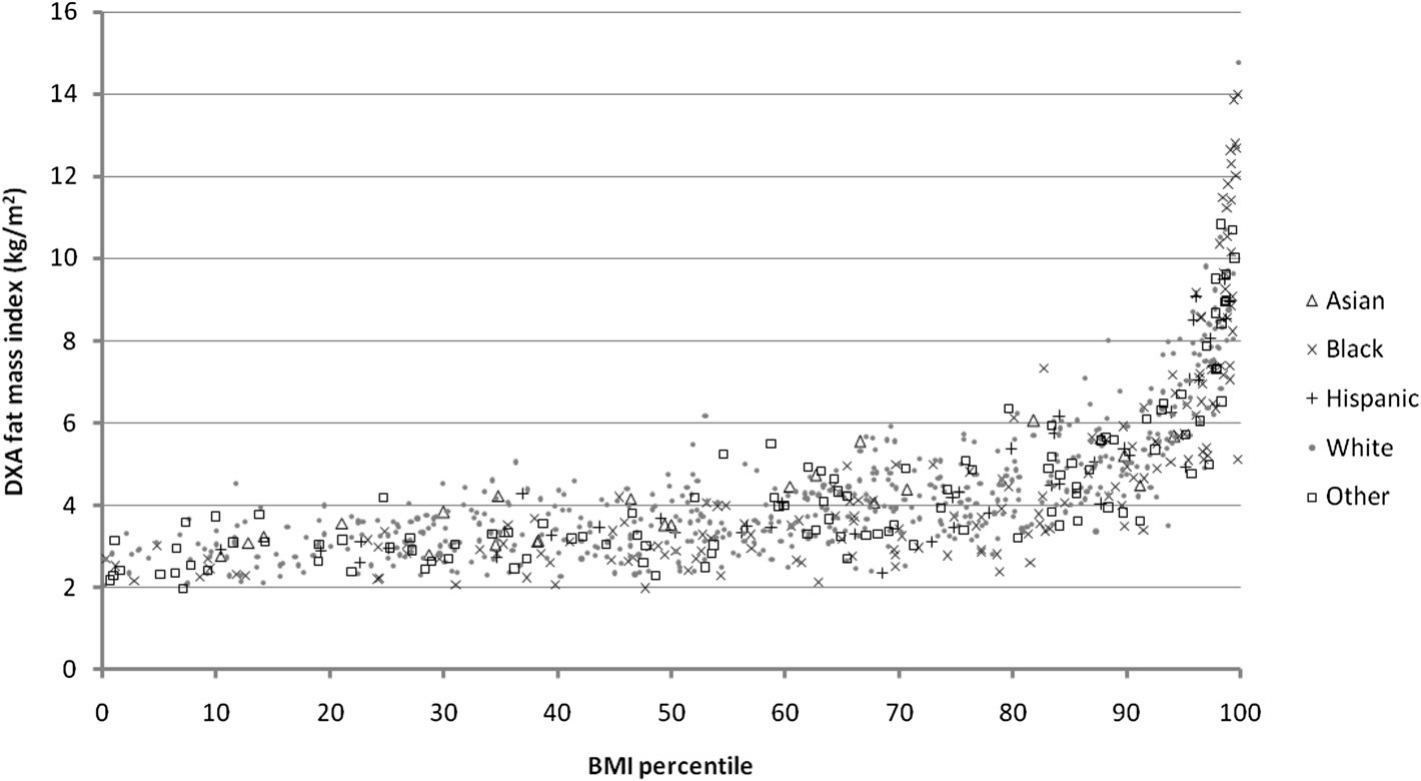
**Age- and sex-specific BMI percentile in relation to DXA fat mass index at 6.5-10.9 years, by race/ethnicity.** Data from 875 participants in Project Viva. BMI percentiles based on CDC growth charts [20].

DXA fat mass increased little for each age-sex-adjusted percentile of BMI until above the 85^th^ percentile (Figure 1).

DXA fat-free mass was highly correlated with bioelectrical impedance fat-free mass (r_s_=0.94) and height (r_s_=0.83) (Table 3). DXA fat-free and fat mass were also correlated with each other to a moderate degree (r_s_=0.54), as were bioimpedance fat and fat-free mass (0.65). BMI had lower correlation with DXA fat-free mass (r_s_=0.69) than with fat mass (r_s_=0.83).

**Table 3:** **Spearman correlations among linear growth and lean body mass measures at 6.5-10.9 years**^**1**^

	**Height**	**Weight**	**BMI**	**Leg length**	**DXA fat-free mass**	**BIA fat-free mass**
	**Spearman R**					
**Height**	1.00	0.80	0.38	0.93	0.83	0.86
**Weight**		1.00	0.84	0.71	0.91	0.93
**BMI**			1.00	0.31	0.69	0.69
**Leg length**				1.00	0.74	0.77
**DXA fat-free mass**					1.00	0.94
**BIA fat-free mass**						1.00

^1^Abbreviations are as follows: *BIA* bioelectrical impedance. Data from 1110 participants in Project Viva. Some participants did not have data for all forms of measurement, so some correlations were among as few as 874 participants. All p-values were significant for these correlations.

BMI-defined overweight plus obesity (≥85^th^ percentile) had a sensitivity of 0.73 and specificity of 0.90 as a predictor of DXA percent body fat cutpoints of ≥30.10% in females and ≥24.63% in males (Table 4). The kappa statistic was 0.63. BMI-defined obesity (≥95^th^ percentile) had a sensitivity of 0.75, specificity of 0.96, and kappa of 0.71 as a predictor of DXA cutpoints of ≥34.00% body fat in girls and ≥29.00% in boys. the area under the ROC curve in this study was 0.90 for overweight plus obesity and 0.94 for obesity.

**Table 4:** Sensitivity and specificity of overweight and obesity: BMI percentile vs. DXA percent body fat cutpoints

**BMI**	**DXA % body fat**							
			**Kappa**	**Sens**	**Spec**	**LR+**	**LR-**	**AUC**
	**Females: ≥30.10% Males: ≥24.63%**^**2**^	**Females: <30.10% Males: <24.63%**^**2**^	0.63	0.73	0.90	7.3	0.31	0.90
**≥85**^**th **^**percentile**^**1**^	169	64						
**<85**^**th **^**percentile**^**1**^	64	578						
	**Females: ≥34.00% Males: ≥29.00%**^**2**^	**Females: <34.00% Males: <29.00%**^**2**^	0.71	0.75	0.96	19.6	0.26	0.94
**≥95**^**th **^**percentile**^**1**^	85	29						
**<95**^**th **^**percentile**^**1**^	29	732						

^1^ Cutoffs defined by CDC growth reference curves [20].

^2^ Based on the prevalence of BMI percentile-defined overweight and obesity in females and males in our dataset. Abbreviations are as follows: *Sens* sensitivity, *Spec* specificity, *LR+* likelihood ratio positive, *LR*- likelihood ratio negative, *AUC* area under the receiver operating characteristic curve.

## Discussion

Among school-aged children enrolled in a US cohort study with research measures of adiposity, we found that BMI, sum of skinfolds, and bioimpedance total fat were all strongly correlated with total fat mass as measured by DXA, which many consider to be a gold standard for field research. Despite concerns that anthropometric measures such as BMI do not distinguish fat mass from lean mass, they were highly correlated with direct measures of adiposity in our study population. These findings suggest that in epidemiologic studies of school-aged children in which DXA is not available, more feasible anthropometric measures such as BMI and skinfold thicknesses are reasonable surrogate measures.

The high correlations we found between DXA and BMI are similar to those in other studies in children [4-7], e.g., BMI and DXA fat mass r=0.85 in a study of 75 American 3–8 year olds vs. r_s_=0.83 in our study) [4]. Our study, however, had more subjects, a mix of different races/ethnicities, and measurements of adiposity using several different methods. The high correlations between DXA and BMI in our study suggest that BMI ranks adiposity relatively well in children.

BMI percentile appeared to correlate more highly with adiposity by DXA fat mass index among overweight and obese children than among normal or underweight children. However, this finding may be due to a wider spread of data among those with higher DXA fat mass than those with less fat mass, as the Bland-Altman plots indicated good agreement between BMI and DXA fat mass index at all levels of adiposity. Similarly, the higher correlations between adiposity measures in black and Hispanic children than white children may be due to differences in the distribution of the data. In our study population, blacks and Hispanics had higher mean and SD in DXA fat mass (9.1 [5.9] kg in blacks, 8.5 [3.9] kg in Hispanics), and wider spread of fat mass values whereas whites clustered together in a narrower range (7.0 [3.0] kg). There may truly be racial/ethnic differences in the relationship between BMI at DXA fat mass in youth, as suggested by Dugas et al., [23] but we were not able to address this in our study. Nevertheless, BMI percentile (or z-score) may be more useful in ranking children’s adiposity in the upper than lower ranges of BMI, as suggested by Freedman et al. [24] Our results extend the findings of Federico et al., who also found a curvilinear association between BMI and percent body fat in 361 Italian children ages 6–12 [25].

Using internally-defined DXA percent body fat cutoffs as a criterion standard for true obesity, we found that BMI-defined obesity had a very high specificity (0.96) and a moderate sensitivity (0.75). Under this scenario, 75% of school-aged children with BMI ≥95^th^ percentile would be correctly classified as obese, and 4% of those with BMI <95^th^ percentile would be incorrectly classified as nonobese. 96% of children whose BMI exceeds the 95^th^ percentile actually had excess adiposity. BMI-defined overweight plus obesity (≥85^th^ percentile) had similar sensitivity (0.73) but slightly lower specificity (0.90). In a review of several studies on this topic, Freedman et al. [24] conclude that BMI ≥95^th^ percentile is a reasonably sensitive (0.7-0.9) and specific (0.95) cutoff for excess adiposity in children, consistent with our results. In addition, the area under the ROC curve was very high (0.90 for overweight plus obesity and 0.94 for obesity), indicating that overall, BMI percentile discriminates effectively between high and low DXA percent body fat.

The sum of skinfold thickness was highly correlated with DXA total fat and was strongly correlated with other measures of adiposity. Previous smaller studies have also found strong associations between reference methods and skinfold thickness using equations [26,27]. Skinfold thicknesses may or may not improve adiposity prediction when combined with BMI [9,28]; in our study, adding the sum of skinfolds variable to a linear regression model of BMI in relation to DXA fat mass index increased the r-squared from 78% to 89%. Our findings demonstrate that with adequate training and thus good reproducibility, skinfold thicknesses constitute valid measures of adiposity among school-age children. Skinfold calipers are relatively inexpensive compared to DXA and are easily transported. Thus skinfold thicknesses could be particularly useful as a direct measure of adiposity in community-based studies.

As in previous studies, we found that bipolar bioelectrical impedance underestimated fat mass and overestimated fat-free mass compared to DXA [4,16-18,29]. This systematic error is concerning for studies using bioelectrical impedance to evaluate absolute fat mass and fat-free mass. However, when the goal is to rank fat mass in a group of children, as in many epidemiologic studies, correlation is more important than absolute levels. In our study, bioimpedance fat mass was highly correlated (r_s_=0.87) with DXA fat mass, consistent with the smaller studies of Eisenmann et al. (r=0.84) [4] and others [16-18]. In addition, we used bipolar bioelectrical impedance. Tetrapolar bioimpedance is likely to be more accurate [30], but experience is limited among young children.

We also examined markers of central adiposity. Trunk fat may be reflective of intra-abdominal fat, which may be more metabolically active than fat stored in other regions of the body. One study of 1987 children and adolescents in Cyprus found that waist circumference and waist-to-height ratio were more highly associated with risk factors for cardiovascular disease like cholesterol and blood pressure than was BMI [12]. In our study, DXA trunk fat was highly correlated with waist circumference, but weakly correlated with waist-to-hip ratio and subscapular-to-triceps ratio. This finding suggests that absolute measures of central adiposity may be more useful than relative ones in ranking fat mass, but the results may also be due to high correlations between these measures and total fat.

When determining which adiposity measurement technique to use, it is important to consider the feasibility and cost of each method in addition to its validity and precision [31]. While DXA differentiates fat mass from lean mass, DXA machines are expensive, not transportable, and require trained personnel, making DXA less feasible than other methods for large studies. Also, DXA emits a small amount of radiation, which may accumulate with repeated measures and may not be acceptable to some research participants. In addition, acquisition time (~5 minutes) may not be feasible for the youngest and most active children. In contrast, BMI and waist circumference can be quickly, cheaply, and safely measured. Measuring skinfold thickness is also inexpensive and directly estimates regional body fat, but accuracy may be poor in obese children [31], and intensive training is required for adequate reproducibility. Bioelectrical impedance, at least the bipolar foot-to-foot measurement, is quick, can be performed almost anywhere using portable equipment, and does not cause radiation exposure, but it still requires equipment that may cost hundreds or thousands of dollars. Since different machines use their own equations to calculate adiposity, output may not be consistent. Further, in our study, the correlation of bioelectrical impedance with DXA was not substantially higher than less expensive anthropometry methods.

This study has several limitations. First, we based our analyses on DXA as a clinical gold standard, whereas a true gold standard is the four-compartment model. Nevertheless, other studies found associations between these two methods to be high (r or r-squared>0.84) among children [2,32]. Second, we did not measure cardio-metabolic biomarkers, which can serve as an external criterion for adiposity measures. Third, this cohort predominantly comprises children of non-low income families, so findings may not be generalizable to other populations. Finally, the age range at the visit was relatively broad, meaning that individuals may have had differences in body composition based on age alone, potentially broadening the distribution of fat mass and increasing correlation coefficients. However, adjusting the correlations for age did not substantially change the correlation coefficients (e.g., DXA and BMI correlation coefficient remained r_s_=0.83), and the broader age range could increase generalizability.

Strengths of our study include the large number of participants, careful measurement by research assistants, and variety of measurement techniques used.

## Conclusions

In school-aged children, BMI, sum of skinfold thicknesses, and other adiposity measures were strongly correlated with DXA fat mass. As anthropometry is less expensive and more feasible than DXA, BMI and skinfolds are reasonable surrogate measures of adiposity in clinical and population studies among children when DXA is not practical. Given the high specificity and predictive value of BMI **≥**95^th^ percentile for DXA-assessed adiposity, clinicians should be confident that using this definition of obesity correctly identifies school-age children with adiposity, even though children below the 95^th^ percentile may also have excess adiposity. Future research should assess whether these findings are consistent in preschool-age children and adolescents. Defining and validating cutoffs for excess adiposity will be important to assess the feasibility of their use in clinical practice.

## Abbreviations

DXA: Dual x-ray absorptiometry; BMI: Body mass index; wt: Weight; ht: Height; BMI Z: Age-sex-adjusted BMI z-score; ROC: Receiver operating characteristic; waist circ: Waist circumference; hip circ: Hip circumference; SS: Subscapular skinfold thickness; TR: Triceps skinfold thickness; BIA: Bioelectrical impedance; Sens: Sensitivity; Spec: Specificity; LR+: Likelihood ratio positive; LR-: Likelihood ratio negative.

## Competing interests

The authors declare that they have no competing interests.

## Authors’ contributions

MWG, EO, and CEB made substantial contributions to study conception and design. SLR and CEB conducted data analysis. All authors helped to interpret the data. CEB drafted the article. All authors revised the manuscript critically and approved the final version of the manuscript.

## Pre-publication history

The pre-publication history for this paper can be accessed here:

http://www.biomedcentral.com/1471-2431/13/99/prepub
